# PEGylation, increasing specific activity and multiple dosing as strategies to improve the risk-benefit profile of targeted radionuclide therapy with ^177^Lu-DOTA-bombesin analogues

**DOI:** 10.1186/2191-219X-2-24

**Published:** 2012-06-09

**Authors:** Simone Däpp, Cristina Müller, Elisa García Garayoa, Peter Bläuenstein, Veronique Maes, Luc Brans, Dirk A Tourwé, Roger Schibli

**Affiliations:** 1 Paul Scherrer Institute, Center for Radiopharmaceutical Sciences ETH-PSI-USZ, Villigen-PSI, 5232, Switzerland; 2 Department of Organic Chemistry, Vrije Universiteit Brussel, Brussels, 1050, Belgium; 3 Department of Chemistry and Applied Biosciences, ETH Zurich, Zurich, 8093, Switzerland

**Keywords:** Gastrin-releasing peptide, Prostate cancer, ^177^Lu, Radionuclide therapy, PEGylation

## Abstract

**Background:**

Radiolabelled bombesin (BN) conjugates are promising radiotracers for imaging and therapy of breast and prostate tumours, in which BN_2_/gastrin-releasing peptide receptors are overexpressed. We describe the influence of the specific activity of a ^177^Lu-DOTA-PEG_5k_-Lys-B analogue on its therapeutic efficacy and compare it with its non-PEGylated counterpart.

**Methods:**

Derivatisation of a stabilised DOTA-BN(7–14)[Cha^13^,Nle^14^] analogue with a linear PEG molecule of 5 kDa (PEG_5k_) was performed by PEGylation of the ϵ-amino group of a β^3^hLys-βAla-βAla spacer between the BN sequence and the DOTA chelator. The non-PEGylated and the PEGylated analogues were radiolabelled with ^177^Lu. *In vitro* evaluation was performed in human prostate carcinoma PC-3 cells, and *in vivo* studies were carried out in nude mice bearing PC-3 tumour xenografts. Different specific activities of the PEGylated BN analogue and various dose regimens were evaluated concerning their therapeutic efficacy.

**Results:**

The specificity and the binding affinity of the BN analogue for BN_2_/GRP receptors were only slightly reduced by PEGylation. *In vitro* binding kinetics of the PEGylated analogue was slower since steady-state condition was reached after 4 h. PEGylation improved the stability of BN conjugate *in vitro* in human plasma by a factor of 5.6. The non-PEGylated BN analogue showed favourable pharmacokinetics already, i.e. fast blood clearance and renal excretion, but PEGylation improved the *in vivo* behaviour further. One hour after injection, the tumour uptake of the PEG_5k_-BN derivative was higher compared with that of the non-PEGylated analogue (3.43 ± 0.63% vs. 1.88 ± 0.4% ID/g). Moreover, the increased tumour retention resulted in a twofold higher tumour accumulation at 24 h p.i., and increased tumour-to-non-target ratios (tumour-to-kidney, 0.6 vs. 0.4; tumour-to-liver, 8.8 vs. 5.9, 24 h p.i.). In the therapy study, both ^177^Lu-labelled BN analogues significantly inhibited tumour growth. The therapeutic efficacy was highest for the PEGylated derivative of high specific activity administered in two fractions (2 × 20 MBq = 40 MBq) at day 0 and day 7 (73% tumour growth inhibition, 3 weeks after therapy).

**Conclusions:**

PEGylation and increasing the specific activity enhance the pharmacokinetic properties of a ^177^Lu-labelled BN-based radiopharmaceutical and provide a protocol for targeted radionuclide therapy with a beneficial anti-tumour effectiveness and a favourable risk-profile at the same time.

## Background

Prostate and breast cancers are the most frequently diagnosed forms of cancer in the USA. Especially in addressing metastatic and small-volume diseases, it is essential to investigate, alongside conventional therapies, alternative treatments, such as peptide receptor radionuclide therapy (PRRT). The fact that certain tumour types overexpress, receptors for peptide-hormones provide the basis for successful use of radiolabelled peptide analogues as tumour tracers in nuclear medicine. The mammalian gastrin-releasing peptide receptor (BN_2_/GRP) [1,2] is particularly overexpressed in several human tumours, including prostate, breast and small-cell lung cancers [3-5]. The tetradecapeptide bombesin (BN) shows high binding affinity for these BN_2_/GRP receptors. Using BN conjugates for specific delivery of radionuclides into the above-mentioned tumours is therefore a promising strategy for diagnostic and therapeutic purposes.

BN analogues, however, present certain problems regarding therapy. They show poor enzymatic stability *in vivo*, which might prevent sufficient localisation at the target site. Furthermore, high accumulation and retention in healthy organs, which express the BN_2_/GRP receptor, increase the risk of side effects. Moreover, kidney toxicity, which was observed and investigated in PRRT with somatostatin analogues in clinical studies [6-8], may also hold true for BN analogues. Finally, several side effects were elicited from intravenous (i.v.) injection of BN agonists in humans. Therefore, a high specific activity of the radiolabelled BN agonist may be important in minimising such undesired effects.

Until now, the research has focused on optimising BN conjugates for nuclear imaging of cancer which overexpresses BN_2_/GRP receptors. Different BN analogues were labelled with diagnostic single-photon emission computed tomography (SPECT) and positron emission tomography (PET) radionuclides, such as ^111^In [9-11], ^99m^Tc [12-15], ^18^ F [16-18], ^68^ Ga [19,20] and ^64^Cu [21], and were evaluated in preclinical studies for their ability to detect BN_2_/GRP receptor-positive lesions. However, only a few radiolabelled BN analogues have been tested in clinics on their diagnostic potential [20,22]. So far, only three BN analogues, AMBA, DOTA-8-AOC-BN(7–14)NH_2_ and DOTA-PESIN, have been rated in preclinical investigations on their potential for radionuclide therapy [23-25]. They were radiolabelled with ^177^Lu (beta-emitter, E_β_ −_max_ 0.497 MeV, half-life of 6.7 days) or with ^213^Bi (alpha-emitter, E_β_ −_max_ 1.423 MeV, E_αmax_ 5.982 MeV, half-life of 45.6 min). The *in vitro* and *in vivo* evaluations of our ^177^Lu-DOTA-Lys-BN analogue (DOTA-β^3^hLys-βAla-βAla-Gln^7^-Trp^8^-Ala^9^-Val^10^-Gly^11^-His^12^-Cha^13^-Nle^14^-NH_2_) showed pharmacokinetic properties which are comparable to that reported for the above-mentioned BN analogues. Therefore, we wanted to improve the radiotherapy-relevant characteristics further by PEGylating ^177^Lu-DOTA-Lys-BN.

Our preclinical study with a series of ^99m^Tc(CO)_3_-labelled PEGylated BN analogues showed that PEGylation is an effective strategy to improve the therapy-relevant characteristics, which include higher tumour uptake, improved tumour retention and lower uptake into non-target tissue. The PEG entity of 5 kDa was established as the optimal PEG size because it improved these features best [26].

**Figure 1:**
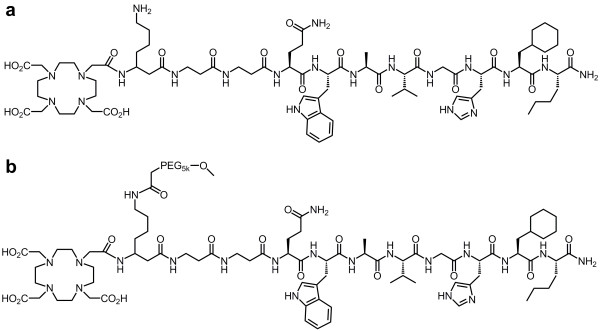
**Chemical structures of (a) DOTA-Lys-BN and (b) DOTA-PEG**_**5k**_**-Lys-BN analogues.**

The BN analogues of the current study were therefore based on one of our stabilised analogues (Gln^7^-Trp^8^-Ala^9^-Val^10^-Gly^11^-His^12^-Cha^13^-Nle^14^-NH_2_) containing a β^3^hLys-βAla-βAla spacer (Figure 1) [14]. The peptide was equipped with a 1,4,7,10-tetraazacyclododecane-1,4,7,10-tetraacetic acid (DOTA) chelator to provide the analogue DOTA-β^3^hLys-βAla-βAla-Gln^7^-Trp^8^-Ala^9^-Val^10^-Gly^11^-His^12^-Cha^13^-Nle^14^-NH_2_ (referred to as DOTA-Lys-BN, Figure 1a). We hypothesised that PEGylating this DOTA-Lys-BN would lead to the same favourable characteristics seen with PEGylated ^99m^Tc-based BN analogues. Derivatisation of the DOTA-Lys-BN analogue with a linear PEG molecule of 5 kDa (PEG_5k_) was performed by PEGylation of the ϵ-amino group of the lysine residue. The resulting PEGylated BN (referred to as DOTA-PEG_5k_-Lys-BN, Figure 1b) as well as the DOTA-Lys-BN were then radiolabelled with ^177^Lu. We chose this radionuclide because it is currently used together with ^90^Y for PRRT with somatostatin analogues on a routine basis in clinics [27,28] and because it proved to be less problematic concerning kidney toxicity in comparison with the ^90^Y-radiolabelled somatostatin analogue [8,27]. Furthermore, application of ^177^Lu allows imaging and PRRT at the same time owing to γ-ray emissions of suitable energy for SPECT, which enables dosimetry calculations and therapy monitoring [29].

In the current study, the new ^177^Lu-labelled DOTA-Lys-BN and DOTA-PEG_5k_-Lys-BN analogues were tested *in vitro* in human prostate carcinoma PC-3 cells and in PC-3 tumour bearing mice. They were compared in order to evaluate the effect of PEGylation on *in vivo* pharmacokinetics and their therapeutic effectiveness. Apart from looking at the anti-tumour efficacy, we also investigated the optimal risk-benefit profile by varying the specific activity of the radiolabelled DOTA-PEG_5k_-Lys-BN analogue and assessed the efficacy of PRRT by varying the number and the interval of the ^177^Lu-DOTA-PEG_5k_-BN doses. For an estimation of potential kidney toxicity, the renal function was monitored with quantitative ^99m^Tc-DMSA scintigraphy.

## Methods

Sources of materials, equipment, peptide synthesis and PEGylation are presented in Additional file 1.

### Statistical analysis

All data are presented as mean ± SD. The *in vivo* data were statistically analysed with a *t* test (Microsoft Excel software). All analyses were 2-tailed and considered as type 3 (two-sample unequal variance); *P* < 0.05 was considered statistically significant.

### ^177^Lu labelling of the DOTA-lys-BN and DOTA-PEG_5k_-lys-BN analogues

For high specific activity labelling, 16 μl of approximately 400 MBq ^177^LuCl_3_ (714.3 GBq/μmol) were added to a mixture of 20 μl ammonium acetate solution (0.5 M, pH 7.5), 84 μl HCl (0.04 M), 5 μl ascorbic acid solution (0.05 M) and 5.6 nmol of BN analogue (high specific, 66 MBq/nmol peptide). The final solution (pH 4.5) was heated at 75 °C for 15 min (Additional file 1: Figure S7). For the ^177^Lu-labelled BN analogues of low specific activity (6.6 MBq/nmol peptide), unlabelled BN analogue was added to the high specific labelling solution to reach the respective concentration.

### Metabolic stability in human plasma

The labelled analogues were incubated with human plasma (final concentration, 10 MBq/0.6 ml) at 37 °C for various time intervals up to 12 days. After incubation, proteins were precipitated with acetonitrile/ethanol (1:1) and TFA (0.1%) and then centrifuged. The supernatant was analysed with RP-high-performance liquid chromatography (HPLC) equipped with a radioactivity detector. The radioactivity chromatograms showed different peaks which corresponded to the intact peptide and the different degradation products. The experiments were performed two times.

### Internalisation and externalisation studies

For internalisation, PC-3 cells at confluence were placed in six-well plates and left to attach overnight. Cells were incubated with the labelled analogues (4 kBq) in culture medium for 0.5, 1, 2, 4 and 24 h at 37 °C. Non-specific binding was determined with 1 μM unlabelled BN(1–14). After the different incubation times, cells were twice washed with cold phosphate buffered saline (PBS) to discard unbound peptide. Surface-bound activity was removed by two 5-min acid washes (50 mM glycin-HCl, 100 mM NaCl, pH 2.8). Afterwards, the cells were washed with cold PBS, and lysed with 1 N NaOH twice. Surface-bound and internalised radioactivities were measured in the gamma counter.

For externalisation, PC-3 cells were incubated with the labelled analogues (60 kBq) in culture medium at 37 °C for 1 h. After incubation, the supernatant was discarded, and the cells were twice washed with cold PBS. The cells were then incubated again at 37 °C in culture medium for 0.5, 1, 2.5, 5 and 24 h. At each time point, the supernatant was collected, the cells twice washed with cold PBS and lysed with 1 N NaOH. The supernatant (released radioactivity) and the cells (bound/internalised radioactivity) were measured in the gamma counter. All experiments were carried out two to three times in triplicate.

### Biodistribution studies

All animal experiments were conducted in compliance with the Swiss animal protection laws and with the ethical principles and guidelines for scientific animal experimentation established by the Swiss Academy of Natural Sciences. Biodistribution studies were performed with 6- to 8-week-old female CD-1 nu/nu mice (20 to 25 g) purchased from Charles River Laboratories (Sulzfeld, Germany). For the induction of tumour xenografts, each mouse received subcutaneously 8 × 10^6^ PC-3 cells in 150 μl culture medium without supplements. The tumours were allowed to grow for at least 3 weeks. On the day of the experiment, the mice (3 to 6 per group) received the radioactive conjugates intravenously. For the biodistribution studies, the mice were injected with different specific activities of the radiolabelled BN analogues (low specific, 6.6 MBq/nmol peptide; high specific, 66 MBq/nmol peptide). Receptor-blocking studies were performed using 100 μg of unlabelled BN(1–14) co-injected with the corresponding radiolabelled BN analogue. At 1, 4 and 24 h post injection (p.i.), the animals were euthanised and dissected. Blood, tumours and various healthy tissues and organs were collected, weighed and examined for radioactivity. Results are expressed as percentage of injected dose per gram of tissue (% ID/g).

### Dose calculation

The absorbed doses to PC-3 tumours and critical organs were calculated from the biodistribution studies (1 MBq/0.1 ml; 0.3 or 3.0 nmol peptide; *n* = 3 per group). Under the assumption of rapid accumulation (uptake at 0 h p.i. corresponds to the uptake at 1 h p.i.), the cumulative radioactivity in each tissue was calculated (MBq/h) taking biologic elimination and physical decay into account up to 24 h p.i. and afterwards only physical decay up to 400 h p.i. The absorbed tumour doses of the mouse experiments were extrapolated from the sphere model doses which were calculated by using the software OLINDA (OLINDA/EXM1.0, Vanderbilt University, Nashville, TN, USA). The *S* values for all other tissues of mice were taken from E Larsson [30]. The absorbed dose (milligray per mega-Bequerel) was calculated by multiplying the area under the curve (AUC) (h; normalised to 1 MBq ID) with the *S* value (mGy/(MBq^☆^s)). The dose (in Gy) was calculated by multiplying the absorbed dose (mGy/MBq) with the amount of radioactivity injected (20 MBq). The AUC-estimate for an adult male was obtained by multiplying the AUC of the mice (MBq/h) with a factor consisting of (total body weight_mouse_/total body weight_adult male_) × organ weight_adult male_. The subsequent dose calculation was performed using the adult male model of the software OLINDA.

### Therapy studies

Therapy studies were conducted in mice bearing PC-3 xenografts. The tumour was assumed to be an ellipsoid, and its volume was calculated with the formula $V_{t}=(\pi /6)LW^{2}$ where *L* represents the longest dimension and *W* the shortest dimension of the tumour. Two weeks post PC-3 inoculation, i.e. the day of the first injection (day 0), the tumours had an average volume of 85 mm^3^. The animals were divided into six groups of six mice (Table 1). The control group received an i.v. injection of PBS only (group A). Another group was injected with unlabelled DOTA-PEG_5k_-Lys-BN at a peptide amount of 3.0 nmol (group B). The treated mice received two equal doses of 20 MBq i.v. either of ^177^Lu-DOTA-PEG_5k_-Lys-BN (groups C to E) or of ^177^Lu-DOTA-Lys-BN (group F) at a peptide amount of 0.3 or 3.0 nmol. The injections were performed either at days 0 and 14 or at days 0 and 7 (Table 1). Body weight and tumour volume of all mice were quantified 3 times per week. The relative volume of tumours *V*_r_ was defined as *V*_r_ = *V*_*t*_/*V*_0_, where *V*_*t*_ was the measurement at time *t* (days after the first injection), and *V*_0_ was the measurement at day 0. If a tumour disappeared, *V*_*t*_ was set to 0. Mice were removed from the study promptly upon fulfilling one or both of the following criteria: a tumour volume of  ≥ 1.5 cm^2^ or total body weight loss of ≥ 15%. Upon euthanasia, tumours were collected and embedded in TissueTek (Sakura Finetek, USA Inc., Torrance, CA, USA) and frozen for autoradiography.

**Table 1:** Therapy protocol: classification of therapy groups and specification of administration of the BN analogues

**Therapy groups**		**BN analogues**	**Dose and peptide amount**	**Injection time**
Mice with PC-3 tumour xenografts	A	PBS	2 × 100 μl	Days 0 and 14
	B	DOTA-PEG_5k_-Lys-BN	2 × 3.0 nmol	Days 0 and 14
	C	^177^Lu-DOTA-PEG_5k_-Lys-BN	2 × 20 MBq/3.0 nmol each	Days 0 and 14
	D	^177^Lu-DOTA-PEG_5k_-Lys-BN	2 × 20 MBq/0.3 nmol each	Days 0 and 14
	E	^177^Lu-DOTA-PEG_5k_-Lys-BN	2 × 20 MBq/0.3 nmol each	Days 0 and 7
	F	^177^Lu-DOTA-Lys-BN	2 × 20 MBq/0.3 nmol each	Days 0 and 14
Mice without PC-3 tumour xenografts	G	PBS	2 × 100 μl	Days 0 and 14
	H	^177^Lu-DOTA-PEG_5k_-Lys-BN	2 × 20 MBq/0.3 nmol each	Days 0 and 14
	I	^177^Lu-DOTA-PEG_5k_-Lys-BN	2 × 20 MBq/3.0 nmol each	Days 0 and 14

### ^99m^Tc-DMSA SPECT/CT imaging studies

Three groups of four mice each (groups G to I, Table 1), which were not xenografted with PC-3 cells, were included in the therapy study for ^99m^Tc-DMSA tests. An untreated control group of mice (group G), a treated group receiving two doses of the ^177^Lu-DOTA-PEG_5k_-Lys-BN analogue at high specific activity (group H) and a treated group of mice getting two doses of the ^177^Lu-DOTA-PEG_5k_-Lys-BN analogue with low specific activity (group I). ^99m^Tc-DMSA scans with SPECT/computed tomography (CT) were obtained 43, 71 and 111 days after therapy, 2 h after i.v. injection of about 30 MBq ^99m^Tc-DMSA. SPECT scans were acquired with anaesthetised mice during 20 min using 15 projections/min. The images were obtained on an X-SPECT-system (Gamma Medica, Inc., Northridge, CA, USA) equipped with a single head SPECT device and a CT device. SPECT data were acquired and reconstructed with LumaGEM (version 5.407, Gamma Medica, Northridge, CA, USA). CT data were acquired with an X-ray CT-system (Gamma Medica) and reconstructed with the software CoBRA (version 4.5.1, Falls Church, VA, USA). SPECT and CT data were combined with the software IDL Virtual Machine (version 6.0, Exelis Visual Information Solutions, Inc., McLean, VA, USA). The images were generated with Amira (version 4.0). Quantification of the amount of radioactivity in a volume of interest over the kidneys was performed with Amira (version 4.0, San Diego, CA, USA). Detected counts in the volume of interest were normalised to 1 MBq ID.

## Results and discussion

### Results

#### *In vitro evaluation*

**Figure 2:**
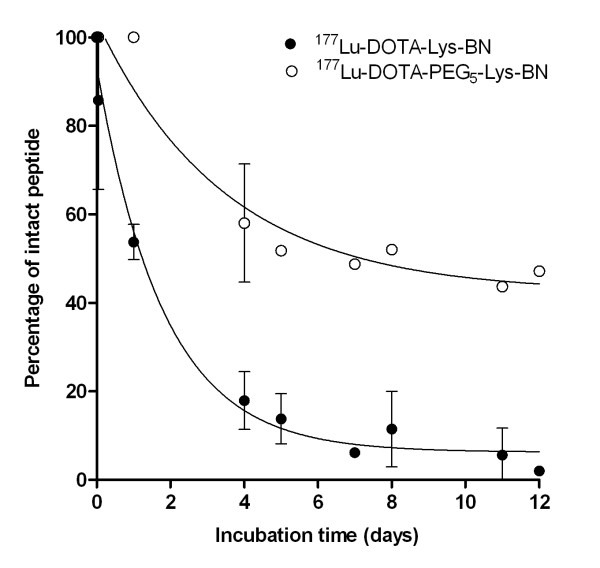
**Metabolic stability of**^**177**^**Lu-DOTA-Lys-BN and**^**177**^**Lu-DOTA-PEG**_**5k**_**-Lys-BN in human plasma.**

The PEGylation of the DOTA-Lys-BN analogue (Additional file 1: Figure S7) as well as the results of the log D and IC_50_ determination are presented in Additional file 1. PEGylation resulted in a slightly increased hydrophilicity of the analogue and in an eightfold higher IC_50_ value compared with that of the non-PEGylated analogue (15.96 ± 4.42 nM vs. 2.03 ± 0.42 nM). The ^177^Lu-DOTA-Lys-BN was rapidly degraded by proteases in human plasma. After 5 days of incubation, it was almost entirely metabolised, and only 13.8 ± 5.7% remained intact. PEGylation resulted in a marked increase in protease stability; 51.8 ± 0.9% of ^177^Lu-DOTA-PEG_5k_-Lys-BN still remained intact after 5 days of incubation and 43.7 ± 0.5% after 11 days. Moreover, the half-life of ^177^Lu-DOTA-Lys-BN in human plasma was 1.2 ± 0.3 days, whereas the half-life of ^177^Lu-DOTA-PEG_5k_-Lys-BN was 6.7 ± 1.4 days (Figure 2).

^177^Lu-DOTA-Lys-BN internalised rapidly into PC-3 cells and reached its maximum within the first hour of incubation (approximately 30%/10^6^ cells). The PEGylated analogue showed a significantly lower and slower internalisation into PC-3 cells. After incubation for 4 h, the internalised fraction was 3.3 ± 1.2%. Externalisation studies revealed 63.1 ± 4.0% of the internalised ^177^Lu-DOTA-Lys-BN externalised within the first 2.5 h. After 24 h, only 13.5 ± 7.2% of the internalised fraction was found in the cells. In contrast, the externalisation of the PEGylated analogue was slower (24.2 ± 1.3% after 24 h).

#### *Biodistribution studies*

The effect of PEGylation was tested *in vivo* in mice with PC-3 tumour xenografts performed with BN conjugates at an injected peptide amount of 0.075 nmol (Table 2). The highest tumour uptake of ^177^Lu-DOTA-Lys-BN (1.88% ID/g) and ^177^Lu-DOTA-PEG_5k_-Lys-BN (3.43% ID/g) was found 1 h p.i., the latter being significantly higher. Thus, the enhanced enzymatic stability induced by PEGylation compensated for the lower receptor affinity of DOTA-PEG_5k_-Lys-BN. Furthermore, the tumour washout was slightly slower for the PEGylated analogue. Thus, 1.04% ID/g was found for the ^177^Lu-DOTA-PEG_5k_-Lys-BN 24 h p.i., whereas only 0.54% ID/g of ^177^Lu-DOTA-Lys-BN remained in the tumour. At 1 h p.i., both analogues showed their highest uptake in the pancreas, which expresses GRP receptors (8.68% ID/g and 9.62% ID/g for ^177^Lu-DOTA-Lys-BN and ^177^Lu-DOTA-PEG_5k_-Lys-BN, respectively). The liver uptake of both ^177^Lu-DOTA-Lys-BN and ^177^Lu-DOTA-PEG_5k_-Lys-BN was low (0.26% ID/g and 0.57% ID/g at 1 h p.i., respectively). Kidney accumulation was higher for the ^177^Lu-DOTA-PEG_5k_-Lys-BN in comparison with that of the non-PEGylated analogue at 1 h p.i. (4.89% ID/g vs. 2.86% ID/g, respectively). The renal clearance, however, was fast for both analogues (1.84% ID/g vs. 1.41% ID/g at 24 h p.i., respectively). The conjugation of a PEG_5k_ entity resulted in a significantly longer blood circulation during 1 h p.i., but both derivatives were completely cleared from blood within 24 h p.i. (Table 2).

**Table 2:** **Biodistribution (0.3 MBq/0.075 nmol) of**^**177**^**Lu-labelled BN analogues in nude mice bearing PC-3 tumour**

	^**177**^**Lu-DOTA-Lys-BN**		^**177**^**Lu-DOTA-PEG**_**5k**_**-Lys-BN**		^**177**^**Lu-DOTA-Lys-BN**	^**177**^**Lu-DOTA-PEG**_**5k**_**-Lys-BN**
**Tissue**	**1 h p.i.**	**24 h p.i.**	**1 h p.i.**	**24 h p.i.**	**1 h p.i. blocked**	**1 h p.i. blocked**
Blood	0.24 ± 0.07	0.00 ± 0.00	1.54 ± 0.33*	0.01 ± 0.00*		
Heart	0.14 ± 0.06	0.01 ± 0.01	0.49 ± 0.05	0.04 ± 0.01		
Lung	0.36 ± 0.08	0.02 ± 0.01	1.10 ± 0.15	0.13 ± 0.02		
Spleen	0.42 ± 0.12	0.18 ± 0.04	0.72 ± 0.02	0.19 ± 0.03		
Kidneys	2.86 ± 0.63	1.41 ± 0.14	4.89 ± 1.33	1.84 ± 0.52		
Pancreas	8.68 ± 1.95	4.27 ± 0.85	9.62 ± 2.39	4.87 ± 1.11	0.49 ± 0.22**	0.98 ± 0.59*
Stomach	0.72 ± 0.19	0.10 ± 0.02	1.12 ± 0.15	0.22 ± 0.07		
Small intestine	1.38 ± 0.41	0.16 ± 0.03	1.18 ± 0.22	0.17 ± 0.12		
Colon	1.64 ± 0.40	0.36 ± 0.09	2.19 ± 0.91	0.45 ± 0.21	0.36 ± 0.07**	0.66 ± 0.14
Liver	0.26 ± 0.09	0.09 ± 0.02	0.57 ± 0.04	0.16 ± 0.09		
Muscle	0.20 ± 0.24	0.01 ± 0.00	0.31 ± 0.06	0.42 ± 0.69		
Bone	0.36 ± 0.24	0.02 ± 0.01	0.70 ± 0.06	0.58 ± 0.44		
Tumour	1.88 ± 0.47	0.54 ± 0.30	3.43 ± 0.63*	1.04 ± 0.04	0.55 ± 0.03*	1.02 ± 0.34**

*P < 0.05; **P < 0.01. Data in percentage of injected dose per gram of tissue (%ID/g), expressed as mean ± SD at 1 and 24 h p.i. (*n* = 3 to 4).

Specificity for GRP receptors could be demonstrated by a co-administration of non-radioactive BN(1–14). Thus, only the uptake in the receptor-expressing tissues such as the pancreas, colon and tumour was markedly reduced (>70%), whereas the inhibition was slightly less effective for the PEGylated BN analogue (Table 2).

**Figure 3:**
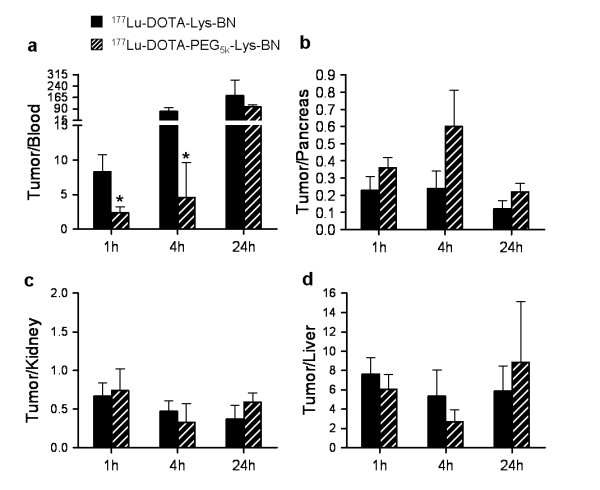
**Tumour-to-non-target ratios.** (**a**) Tumour-to-blood, (**b**) tumour-to-pancreas, (**c**) tumour-to-kidney and (**d**) tumour-to-liver ratios.

The tumour-to-non-target ratios were similar for both derivatives. The initial longer circulation time of ^177^Lu-DOTA-PEG_5k_-Lys-BN, however, resulted in lower tumour-to-blood ratios at 1 and 4 h p.i. compared with that of the non-PEGylated compound. ^177^Lu-DOTA-PEG_5k_-Lys-BN showed a twofold increase in the tumour-to-pancreas ratio at all time points and in tumour-to-kidney and tumour-to-liver ratios at 24 h p.i. (Figure 3).

**Figure 4:**
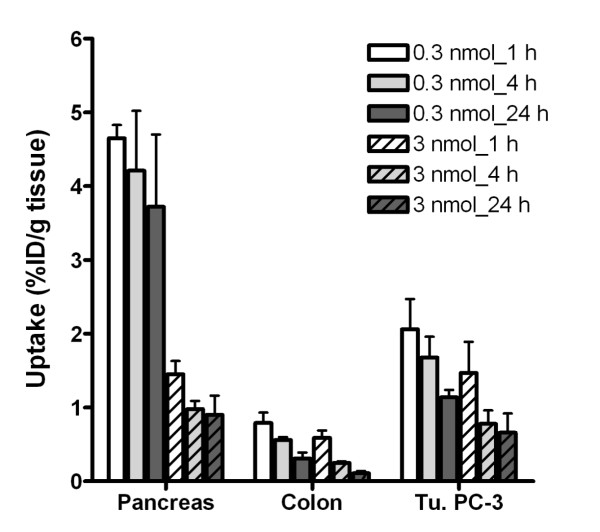
**Influence of the amount of injected peptide on the biodistribution of**^**177**^**Lu-DOTA-PEG**_**5k**_**-Lys-BN.**

 In additional biodistribution studies, two ligand doses of the ^177^Lu-DOTA-PEG_5k_-Lys-BN at a peptide amount corresponding to the amount that was injected in the therapy studies (high specific, 0.3 nmol; or low specific, 3.0 nmol peptide injected per mouse) were administered. This showed that the uptake in the receptor-expressing tissues such as the pancreas and colon was markedly reduced by applying a high amount of PEGylated peptide (3.0 nmol). The tumour uptake was also reduced by 29% and 42% at 1 h p.i. and 24 h p.i. after injection of a high amount of peptide (Figure 4, Table 3). In comparison, the tumour-to-blood, tumour-to-liver, tumour-to-kidney and tumour-to-muscle ratios were approximately twofold higher at all time points if ^177^Lu-DOTA-PEG_5k_-Lys-BN was injected at a low molar amount of peptide (0.3 nmol). The only ratios which revealed a higher value with a high amount of peptide (3.0 nmol) were the tumour-to-pancreas ratios (Table 3).

**Table 3:** **Biodistribution (1 MBq/0.3 or 3.0 nmol) of**^**177**^**Lu-DOTA-PEG**_**5k**_**-Lys-BN analogue in nude mice bearing PC-3 tumours**

	^**177**^**Lu-DOTA-PEG**_**5k**_**-Lys-BN**			
	**1 h p.i.**		**24 h p.i.**	
**Tissue**	**0.3 nmol**	**3.0 nmol**	**0.3 nmol**	**3.0 nmol**
Blood	0.51 ± 0.09	0.77 ± 0.11*	0.02 ± 0.00	0.02 ± 0.00
Heart	0.22 ± 0.05	0.31 ± 0.09	0.04 ± 0.01	0.05 ± 0.01
Lung	0.92 ± 0.59	2.03 ± 1.64	0.44 ± 0.32	0.58 ± 0.36
Spleen	0.45 ± 0.11	0.39 ± 0.05	0.40 ± 0.06	0.21 ± 0.04
Kidneys	3.11 ± 0.42	3.92 ± 0.59	1.59 ± 0.42	2.12 ± 0.21
Pancreas	4.65 ± 0.18**	1.45 ± 0.18	3.72 ± 0.98*	0.90 ± 0.26
Stomach	0.42 ± 0.09	0.89 ± 0.65	0.16 ± 0.03	0.14 ± 0.10
Small intestine	0.55 ± 0.02	0.78 ± 0.39	0.20 ± 0.04*	0.10 ± 0.02
Colon	0.79 ± 0.14	0.59 ± 0.10	0.31 ± 0.08*	0.11 ± 0.03
Liver	0.50 ± 0.06	0.64 ± 0.07	0.51 ± 0.05	0.46 ± 0.02
Muscle	0.17 ± 0.03	0.20 ± 0.05	0.02 ± 0.01	0.02 ± 0.00
Bone	0.21 ± 0.03	0.31 ± 0.03	0.23 ± 0.03	0.22 ± 0.03
Tumour	2.06 ± 0.41*	1.47 ± 0.42	1.14 ± 0.10	0.66 ± 0.26
Tumour-to-blood	4.08 ± 0.54	1.89 ± 0.42	62.46 ± 3.58	36.24 ± 13.73
Tumour-to-liver	4.11 ± 0.67	2.28 ± 0.60	2.78 ± 0.21	1.47 ± 0.56
Tumour-to-kidney	0.67 ± 0.14	0.38 ± 0.13	0.62 ± 0.07	0.33 ± 0.16
Tumour-to-muscle	12.26 ± 4.01	7.20 ± 0.90	55.89 ± 32.86	27.27 ± 11.92
Tumour-to-pancreas	0.44 ± 0.08	1.01 ± 0.23	0.28 ± 0.08	0.62 ± 0.22

*P < 0.05.^;^ **P < 0.001. Data in percentage of injected dose per gram of tissue (%ID/g), expressed as mean ± SD at 1 and 24 h p.i. (*n* = 3).

#### *Dose calculation*

After applying ^177^Lu-DOTA-PEG_5k_-Lys-BN at a low molar amount of peptide (0.3 nmol) in a single dose, the absorbed doses were calculated to be 0.36 Gy/MBq for the murine kidney, 0.002 Gy/MBq for the blood, 0.02 Gy/MBq for the pancreas and 0.19 Gy/MBq for the tumour. After the application of ^177^Lu-DOTA-PEG_5k_-Lys-BN at a high amount of peptide (3.0 nmol), however, the absorbed doses to the murine kidney, blood, pancreas and tumour were calculated to be 0.50, 0.002, 0.006 and 0.11 Gy/MBq, respectively. The estimate for an adult male resulted in absorbed doses to the kidney, blood and pancreas of 9.4, 0.4 and 21.3 Gy/GBq (0.3 nmol) and 12.3, 0.4 and 5.2 Gy/GBq (3.0 nmol), respectively.

#### *Therapy studies*

**Figure 5:**
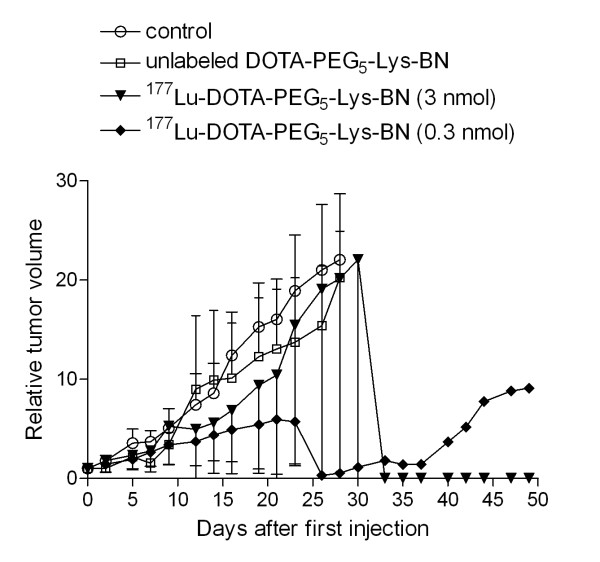
**Therapeutic effect of**^**177**^**Lu-DOTA-PEG**_**5k**_**-Lys-BN in mice with PC-3 tumour xenografts.** The graph shows the comparison of treatment with high specific activity (2 × 20 MBq/0.3 nmol peptide each; group D) and low specific activity (2 × 20 MBq/3.0 nmol peptide each; group C). Besides, it shows the effect of unlabelled DOTA-PEG_5k_-Lys-BN (2 × 3.0 nmol peptide; group B) on tumour growth. Data are expressed as the volume of tumour relative to the volume in the same animal immediately before the first injection (mean ± SD of six animals).

**Figure 6:**
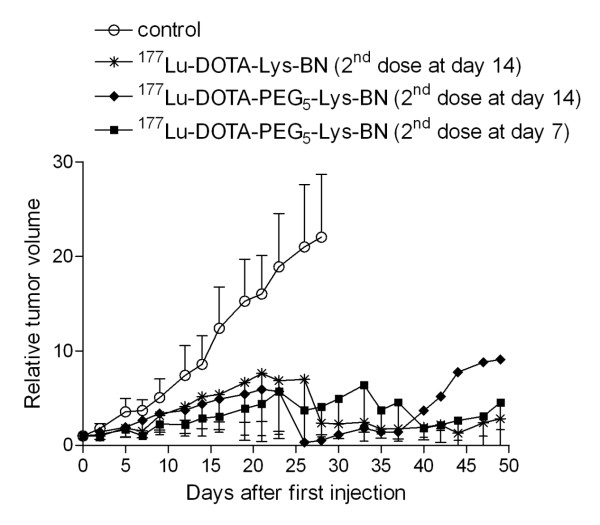
**Therapeutic effect of the**^**177**^**Lu-labelled BN analogues.** Peptides were labelled with high specific activity (2 × 20 MBq/0.3 nmol peptide each) and injected in mice with PC-3 tumour xenografts. The graph shows the comparison of treatment with the PEGylated (group D) and the non-PEGylated (group F) ^177^Lu-labelled BN analogues. Besides, it shows the influence of timing the second dose (second dose at day 7 vs. day 14; group E vs. group D). Data are expressed as the volume of tumour relative to the volume in the same animal immediately before the first injection (mean ± SD of six animals).

The therapy study was performed according to the protocol shown in Table 1. In total, 48 mice were included and divided into six groups of mice bearing PC-3 tumours (groups A to F; *n* = 6) and three additional groups of mice without tumours (groups G to I; *n* = 4). All groups of mice which received the ^177^Lu-labelled BN analogues (C to F) clearly showed a reduction of tumour growth in comparison with groups A and B which received only PBS or unlabelled BN analogue. The treatment with the non-PEGylated ^177^Lu-labelled BN analogue (0.3 nmol; group F) significantly decreased the PC-3 tumour growth rate with respect to that of group A and exhibited an inhibition of 53% 3 weeks after the first dose (Figures 5 and 6). The tumour growth inhibition was higher (63%) with the ^177^Lu-DOTA-PEG_5k_-Lys-BN analogue of high specific activity (group D). However, the ^177^Lu-DOTA-PEG_5k_-Lys-BN analogue of low specific activity (group C) exhibited only an inhibition of 36%. The most effective tumour growth inhibition of 73% (3 weeks after the first dose) was observed when the second dose of the PEGylated BN analogue of high specific activity was applied 7 days after the first dose (group E; Figure 6). Mice of group B did not show an increased tumour growth with respect to group A (Figure 5), although BN agonists are known to have mitogenic characteristics.

#### ^*99m*^*Tc-DMSA SPECT/CT imaging studies*

Forty-three days after therapy, the renal ^99m^Tc-DMSA uptake of the treated animals (group H) receiving the radiotracer of high specific activity was 76,397 counts/kidney, whereas the uptake of the treated animals receiving the radiotracer of low specific activity (group I) was 74,949 counts/kidney. Seventy-one days after therapy, there was no significant difference in the renal ^99m^Tc-DMSA uptake between groups G, H and I (51,344, 57,147 and 47,692 counts/kidney, respectively); 111 days after therapy, there was also no significant difference in the renal ^99m^Tc-DMSA uptake between these three groups of mice.

## Discussion

So far, only three optimised BN analogues, DOTA-8-AOC-BN(7–14)NH_2_, AMBA (DO3A-CH_2_CO-8-aminooctanoyl-Gln-Trp-Ala-Val-Gly-His-Leu-Met-NH_2_) and DOTA-PESIN (DOTA-15-amino-4,7,10,13-tetraoxapentadecanoic acid-Gln-Trp-Ala-Val-Gly-His-Leu-Met-NH_2_), have been evaluated for PRRT [23-25]. These compounds were radiolabelled with the therapeutic radioisotopes ^177^Lu or ^213^Bi and showed anti-tumour effectiveness in mice with PC-3 xenografts. Regarding *in vitro* evaluation and biodistribution data, our ^177^Lu-DOTA-Lys-BN analogue showed pharmacokinetic properties which are comparable to those of the above-mentioned BN analogues, except for the higher tumour uptake and the better retention profile of AMBA and DOTA-PESIN. Therefore, we wanted to improve the radiotherapy relevant characteristics further by PEGylating ^177^Lu-DOTA-Lys-BN.

*In vitro*, time-dependent cell uptake and internalisation showed slower binding kinetics for the PEGylated BN analogue. These findings are in line with the results of PEGylating other biomolecules reported in the literature [31]. PEG is also reported to affect target association and dissociation rates of antibody fragments negatively [32]. These aspects may apply to our ^177^Lu-DOTA-PEG_5k_-Lys-BN and explain why binding affinity of this analogue *in vitro* was slightly reduced ( Additional file 1), the steady state was reached later, and the total cell binding was lower in comparison with that of the non-PEGylated counterpart.

Previously, we could confirm that PEGylation improves the stability of BN toward enzymatic degradation [26]. In the case of DOTA-Lys-BN, conjugation of PEG_5k_ also led to a considerable increase in stability *in vitro* (Figure 2). The half-life (*t*_1/2_) of ^177^Lu-DOTA-PEG_5k_-Lys-BN in human plasma was 5.6-fold higher in comparison with that of non-PEGylated ^177^Lu-DOTA-Lys-BN. In comparison to ^177^Lu-AMBA, which is more stable in human plasma (*t*_1/2_ = 38.8 h) [23] than ^177^Lu-DOTA-PESIN (*t*_1/2_ = 8.4 h) [25], the *in vitro* half-life of ^177^Lu-DOTA-Lys-BN in human plasma (*t*_1/2_ = 28.8 h) was in the same range but was markedly higher with the PEGylated BN analogue (*t*_1/2_ = 160.8 h).

The biodistribution data, in which 0.002 nmol of ^177^Lu-AMBA and ^177^Lu-DOTA-8-AOC-BN(7–14) (HPLC purified) was injected per mouse [23], and the data of ^177^Lu-DOTA-PESIN (0.2 nmol peptide) [25] were compared with our biodistribution data, in which 0.075 nmol of the ^177^Lu-labelled BN analogues were injected. This 0.075 nmol is the nearest possible approximation to the 0.002 nmol without HPLC purification, which is desired in clinics. In comparison with ^177^Lu-AMBA, our ^177^Lu-DOTA-Lys-BN showed an approximately fourfold lower kidney uptake 1 h p.i., whereas the kidney uptake of the ^177^Lu-DOTA-PEG_5k_-Lys-BN analogue was 2.3-fold lower at 1 h p.i. Both compounds showed a faster clearance from the kidneys within 24 h p.i. Kidney accumulation and washout of our ^177^Lu-DOTA-Lys-BN and ^177^Lu-DOTA-PEG_5k_-Lys-BN were comparable to those of ^177^Lu-DOTA-PESIN (3.8 ± 0.34%ID/g at 1 h p.i.), even though Gelofusine and polyglutamic acid were co-administered with ^177^Lu-DOTA-PESIN for the reduction of renal uptake [25]. Furthermore, the GI uptake was much lower with ^177^Lu-DOTA-Lys-BN and ^177^Lu-DOTA-PEG_5k_-Lys-BN at 1 and 24 h p.i. compared with that in ^177^Lu-AMBA (11.2%ID and 5.8% ID, respectively) and ^177^Lu-DOTA-8-AOC-BN(7–14) (9.7%ID and 1.7% ID, respectively) [23]. However, the significantly higher blood level at 1 h p.i. after PEGylation might cause higher bone marrow toxicity and could therefore be a potential drawback of PEGylation.

^177^Lu-DOTA-PEG_5k_-Lys-BN showed significantly higher tumour uptake at 1 h p.i. in comparison with the non-PEGylated counterpart. The higher enzymatic stability as well as the longer blood circulation may have compensated for the slower binding kinetics and the lower receptor affinity of DOTA-PEG_5k_-Lys-BN. In order to compare the cumulative radioactivity over 24 h of each conjugate in the tumour, the AUC value of ^177^Lu-DOTA-Lys-BN was arbitrarily set to 1. The comparison showed a relative AUC value of 1.6 (*P* < 0.0006) for ^177^Lu-DOTA-PEG_5k_-Lys-BN.

The second hypothesis that PEGylation prolongs the tumour retention was also proven. Even though PEGylation lowered the tumour washout only slightly between 1 and 24 h p.i., there was more ^177^Lu-DOTA-PEG_5k_-Lys-BN retained in the tumour between 0 and 24 h p.i. The extended tumour retention for the ^177^Lu-DOTA-PEG_5k_-Lys-BN might be explained by the improved enzymatic stability of the peptide derivative, and the extended retention might be due to the enhanced permeation and retention in the tumour. On the basis of the biodistribution data with ^177^Lu-AMBA [23] and ^177^Lu-DOTA-PESIN [25], both BN analogues showed higher tumour uptakes (6.35 ± 2.23% ID/g and 11.6 ± 1.4%ID/g at 1 h p.i., respectively) and better retention profiles than our ^177^Lu-labelled BN analogues. However, compared to ^177^Lu-DOTA-8-AOC-BN(7–14) [23] (2.84 ± 1.65% ID/g at 1 h p.i.), our ^177^Lu-DOTA-Lys-BN analogue showed a similar tumour uptake, but the uptake of ^177^Lu-DOTA-PEG_5k_-Lys-BN was higher. This comparison, however, must be looked at with due care because the study designs differ insofar as different peptide amounts were injected.

The third hypothesis, i.e. that PEGylation improves tumour-to-non-target ratios, could partially be confirmed. The tumour-to-non-target ratios were rather similar for both derivatives. However, in comparison with the non-PEGylated BN analogue, the ^177^Lu-DOTA-PEG_5k_-Lys-BN analogue exhibited a higher tumour uptake and a prolonged tumour retention which resulted in increased tumour-to-pancreas ratios at all time points and in higher tumour-to-liver and tumour-to-kidney ratios at 24 h p.i. (Figure 3).

Alongside PEGylation, the influence of the specific activity on biodistribution was evaluated. ^177^Lu-DOTA-PEG_5k_-Lys-BN injected at two different peptide amounts corresponding to the amount that was injected in the therapy studies (0.3 or 3.0 nmol, respectively) affected the uptake into receptor-expressing tissues. The amount of 0.3 nmol was selected to approximate the 0.22 nmol of the AMBA therapy study because these amounts of 0.3 nmol have proven to be the limit for high specific labelling, i.e. the labelling is reproducible without any loss in yield. The amount of 3.0 nmol however was selected because a preliminary study (data not presented) had suggested that peptide amounts in this range markedly reduce the uptake into non-target receptor positive tissues. In comparison with a low peptide amount, applying a high peptide amount resulted in a marked reduction in pancreas and colon uptake which would lower the risk of radiotoxic side effects induced by radionuclide therapy (Figure 4). However, the cumulative radioactivity in the tumour was significantly higher with a low peptide amount. The dosimetry showed that the absorbed dose into the tumour was 1.7-fold higher with the radiotracer of high specific activity, which would presumably indicate a higher anti-tumour effect. Furthermore, a lower accumulation in the kidneys within 24 h p.i. was observed with ^177^Lu-DOTA-PEG_5k_-Lys-BN at a low amount of peptide (Table 3), which would indicate a reduced risk of nephrotoxicity induced by radionuclide therapy. Thus, the incidence of BN-related toxicity after i.v. injection could be reduced using a low amount of peptide.

The radionuclide therapy studies (Table 1) showed a higher anti-tumour effectiveness with ^177^Lu-DOTA-PEG_5k_-Lys-BN (group D) compared with ^177^Lu-DOTA-Lys-BN (group F) (63% vs. 53% inhibition 3 weeks after the first dose, respectively; Figure 6). This is in accordance with the biodistribution data, which showed a higher tumour uptake and retention after PEGylation (Table 2). As comparative time point, we chose 3 weeks after the first dose, in order to evaluate the effectiveness of the different therapy protocols. This is the latest time point before several mice had to be euthanised upon fulfilling the endpoint criteria. Therefore, an interpretation after 3 weeks is not reliable since the groups represent only individual mice (Figures 5 and 6).

The therapy studies, in which the specific activity was varied (group C vs. group D), resulted in a markedly higher therapeutic efficiency when ^177^Lu-DOTA-PEG_5k_-Lys-BN was applied at high specific activity (63% vs. 36% inhibition 3 weeks after the first dose). The lower tumour accumulation of ^177^Lu-DOTA-PEG_5k_-Lys-BN of low specific activity resulted in a proportionally faster tumour growth. We could demonstrate that the reduced efficacy is not caused by the tumour growth-promoting effect of the higher peptide amount since unlabelled DOTA-PEG_5k_-Lys-BN (group B) did not induce tumour growth compared with the control group (Figure 5). These results are in line with previous observations reported in the literature [25]. The high specific therapy, as we have seen, was more efficient than the low specific, which is in accordance with the biodistribution studies which demonstrate that the uptake in GRPR-expressing tissues is highest for the lower peptide dose and is reduced with the higher peptide dose. This phenomenon is considered to be the result of partial saturation of receptors in the target tissues at higher peptide doses.

Furthermore, it can be assumed that an increase in specific activity would achieve at least the same therapeutic efficiency as low specific activity, but the dosage injected would be lower.

Preliminary therapy studies (Additional file 1: Figure S8), as expected, showed that the administration of two doses (2 × 20 MBq = 40 MBq) was more effective in tumour growth inhibition than application of a single dose (20 MBq). As shown with *in vitro* autoradiography (Additional file 1: Figure S9), there was no long-lasting down-regulation of BN_2_/GRP receptors in the tumour after treatment, which suggests that it is sensible to apply a multiple dosage. Therefore, two different two-dose regimens were evaluated in the current therapy studies. Applying the second dose at day 14 was chosen to match the AMBA therapy study. The preliminary study showed that the tumour started to grow after 14 days regardless of the second injection. Since the cause for this might have been that the tumour was already too large to respond to the treatment, the second application was introduced at day 7 in order to hit the tumour in an earlier state. The therapeutic efficiency was increased even further when the second dose of ^177^Lu-DOTA-PEG_5k_-Lys-BN (group E) was applied 7 days after the first dose instead of 14 days (73% vs. 63% inhibition at day 21) (group D).

Comparing our study with the therapy studies with ^177^Lu-AMBA [23], ^177^Lu-DOTA-PESIN [25] (2 × 28 MBq, 0.2 nmol) and ^177^Lu-DOTA-8-AOC-BN(7–14) [24], we found the tumour growth inhibition with our PEGylated BN analogue to be lower than with AMBA (approximately 73% vs. approximately 82%) but higher than with DOTA-PESIN (approximately 73% vs. approximately 45%) and roughly the same as with ^177^Lu-DOTA-8-AOC-BN(7–14) (approximately 73% vs. approximately 79%) 3 weeks after the first dose. However, such a comparison is not fully conclusive since these therapy studies differ in tumour size at the beginning of therapy, injected peptide amount, administered radiation dose and injection interval.

In order to assess the risk for nephrotoxicity related to radionuclide therapy, a rough dosimetric estimate for an adult male was performed based on the biodistribution, in which 0.3 or 3.0 nmol of the ^177^Lu-DOTA-PEG_5k_-Lys-BN analogue were applied. This estimate implies that an administration of approximately 2 GBq of either low or high specific ^177^Lu-DOTA-PEG_5k_-Lys-BN analogue would result in absorbed kidney doses of approximately 18.8 or 24.6 Gy, respectively. These doses would not exceed the acceptable safe limit of 23 to 27 Gy [33]. The administration of 2 GBq would supposedly be necessary to reach a tumour dose of 50 Gy (supposed that the absorbed dose into the pancreas corresponds to the tumour dose), which is needed for treatment as external beam radiation therapy and brachytherapy data suggest [34-36]. A further step in the risk assessment was ^99m^Tc-DMSA scintigraphy which showed that there was no kidney damage in the mice treated with high or low specific ^177^Lu-DOTA-PEG_5k_-Lys-BN analogue (group H and I) since there was no significant difference in renal ^99m^Tc-DMSA uptake of control and treated mice. Besides, serum analysis confirmed the absence of renal toxicity (Additional file 1).

## Conclusions

PEGylation, increasing the specific activity of the radiolabelled bombesin analogue and shortening the injection interval proved to be effective strategies to enhance the radiotherapeutic efficacy and to provide a favourable risk-profile at the same time. Tumour targeting was optimised and tumour retention was prolonged with the ^177^Lu-DOTA-PEG_5k_-Lys-BN analogue of high specific activity. The estimate of the absorbed doses for an adult male implied that the absorbed kidney doses would lie below the threshold of kidney damage. Taking the positive features into account, which have been observed in this study, we believe that PEGylation of small molecular weight radiopharmaceuticals is an efficient strategy to improve their potential for a successful application in targeted radionuclide therapy.

## Competing interests

The authors declare that they have no competing interests.

## Authors’ contributions

SD participated in the whole study and drafted the manuscript. CM participated in the studies concerning stability, biodistribution, therapy and ^99m^Tc-DMSA SPECT/CT imaging, proofread the manuscript and made suggestions. EGG participated in the biodistribution studies. PB contributed to the dose calculation. VM, LB and DAT carried out the peptide synthesis. RS supervised the study and proofread the manuscript. All authors read and approved the final manuscript.

## Supplementary Material

Additional file 1 **Synthetic details of the PEGylation of the DOTA-Lys-BN analogue, experimental details of the octanol/PBS partition coefficient (log D) determination, details of the apparent receptor affinity (IC_50_) and serum analyses, results of the preliminary therapy study and the results of the *in vitro* autoradiography of tumour sections are presented in the Additional file.** References [23,25,37-39] are included in the Additional file 1. Click here for file
