# Supplementary material for: PEGylation, increasing specific activity and multiple dosing as strategies to improve the risk-benefit profile of targeted radionuclide therapy with 177Lu-DOTA-bombesin analogues
Source: EJNMMI Res. 2012 Jun 9;2:24. doi: 10.1186/2191-219X-2-24 (PMC3478187; doi:10.1186/2191-219X-2-24)
Supplement: Additional file 1 — Synthetic details of the PEGylation of the DOTA-Lys-BN analogue, experimental details of the octanol/PBS partition coefficient (log D) determination, details of the apparent receptor affinity (IC50) and serum analyses, results of the preliminary therapy study and the results of the in vitro autoradiography of tumour sections are presented in the Additional file. References [23,25,37-39] are included in the Additional file 1. [file 2191-219X-2-24-S1.doc]

**SUPPLEMENTAL DATA**

**General**

Methoxypolyethylene glycol succinimidyl ester (MeO-PEG-NHS; M_W_ (average)=5 kDa) was obtained from Iris Biotech (Marktredwitz, Germany). The DOTA-Lys-BN analogue was synthesized on a Rink amide resin using the Fmoc peptide synthesis strategy as previously described [1]. Na[^99m^TcO_4_] was eluted from a ^99^Mo/^99m^Tc generator (Mallinckrodt-Covidien, Petten, The Netherlands) by using 0.9% saline. The dimercaptosuccinic acid (DMSA) kits (Mallinckrodt-Covidien, Petten, The Netherlands) were radiolabelled with ^99m^Tc according to the provided instructions (200 MBq/ml). ^177^LuCl_3_ was purchased from Isotope Technologies Garching GmbH (Munich, Germany). All chemicals not mentioned above were obtained from either Fluka (Buchs, Switzerland) or Merck (Dietikon, Switzerland).

The purification and analysis of the labelled peptides were performed with a Macherey-Nagel CC Nucléosil 100-5 C_18_ reverse-phase column (10 μm, 250x4.6 mm). The separation was performed with HPLC solvents consisting of 25 mM ammonium acetate pH 4.5 and methanol. The HPLC analyses of the radiolabelled peptides were performed with an acetonitrile-water gradient.

The human prostate adenocarcinoma cell line PC-3 was obtained from the European Collection of Cell Culture (CRL-1687, ECACC; Salisbury, England). The cells were maintained in DMEM GLUTAMAX-I supplemented with 10% FCS, 100 IU/ml penicillin G sodium, 100 μg/ml streptomycin sulphate and 0.25 μg/ml amphotericin B. The cells were incubated at 37°C in an atmosphere containing 5% CO_2_ and twice weekly subcultured after detaching with trypsin/EDTA (0.25%).

**PEGylation of the DOTA-Lys-BN analogue**

Twenty μl (10 mM) of methoxypolyethylene glycol succinimidyl ester (MeO-PEG_5k_-NHS; M_W_ (average)=5 kDa) dissolved in 50 mM Na_2_B_4_O_7_ buffer pH 8.0 were mixed with 40 μl (1 mM) of the analogue DOTA-Lys-BN dissolved in distilled water and incubated for 30 min at room temperature. Then, additional 20 μl (5 mM) of MeO-PEG_5k_-NHS dissolved in 50 mM Na_2_B_4_O_7_ buffer pH 8.0 were added five times with a 15-min interval. The solvents were removed by evaporation and the final product was obtained as a lyophilized crude powder which was then dissolved in 40 μl twice distilled water before labelling with ^177^Lu (Supplemental Figure 7). The DOTA-PEG_5k_-Lys-BN conjugate was obtained in yields above 96%. MALDI-TOF spectrum gave a peak with maximum counts at *m/z* 1632.9 for [MH]^+^ (DOTA-Lys-BN). Because of the polydispersity of PEG, the MALDI-TOF spectrum gave a broad group of peaks with maximum counts at *m/z* 6309.2 for [MH]^+^ (DOTA-PEG_5k_-Lys-BN).


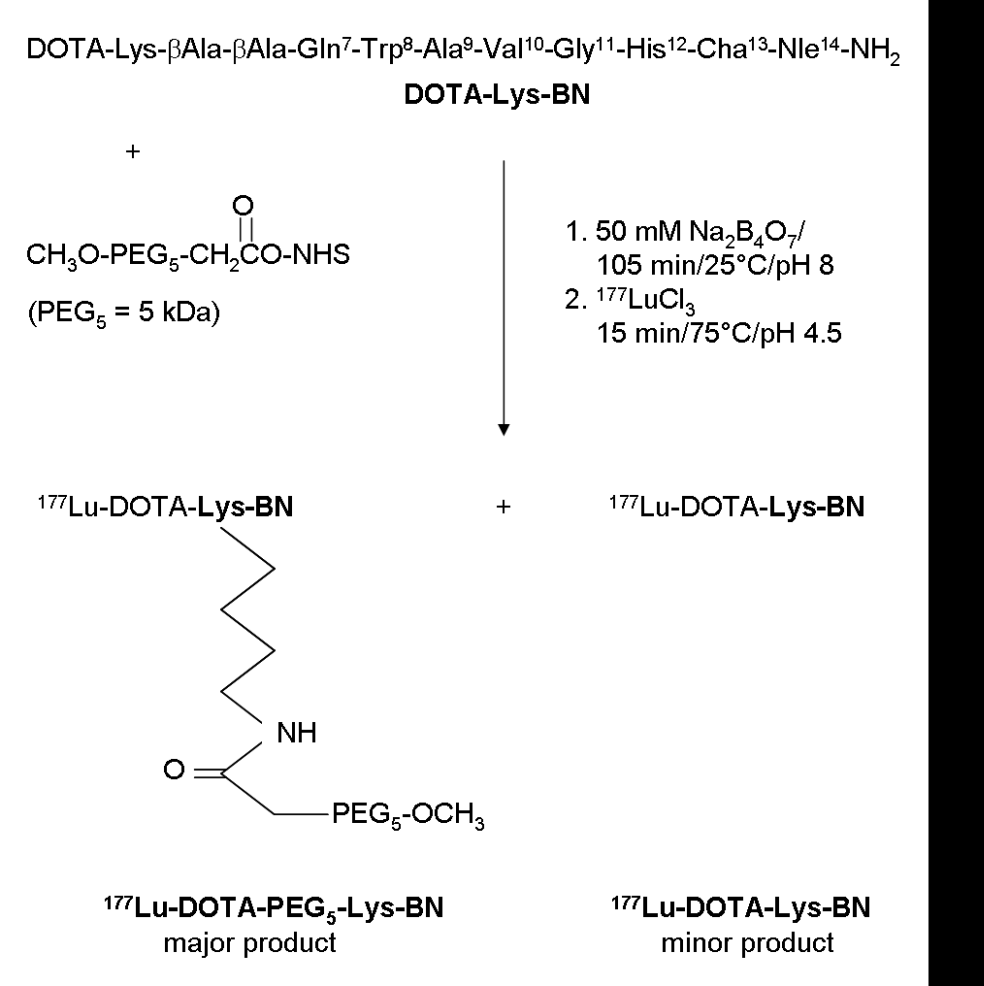


**Supplemental Figure 7** PEGylation and radiosynthesis of the ^177^Lu-DOTA-Lys-BN analogue.

**Octanol/PBS partition coefficient (log D)**

*Material & Methods:* The octanol/PBS partition coefficients were determined at pH 7.4. Five μl containing 30 kBq of the radiolabelled compounds in phosphate buffered saline (PBS) were added to a vial which contained 1.2 ml of 1-octanol and PBS (1:1). After vortexing for 1 min, the vial was centrifuged (5 min, 10 000 rpm) to ensure complete separation of layers. Then, 40 μl of each layer were taken in a preweighed vial and measured in a gamma counter (Packard Canberra Cobra II, Meriden, USA). Counts per weight unit of sample were calculated and log D values were determined as described by Pillarsetty et al. [2] using the formula log_10_D=log_10_ (counts in 1 g of octanol/counts in 1 g of PBS). The experiments were performed in sextuplicate.

*Results:* The log D value determined for ^177^Lu-DOTA-Lys-BN was -2.18±0.07. PEGylation resulted in a slightly increased hydrophilicity of the analogue with a log D value of -2.37±0.38 for ^177^Lu-DOTA-PEG_5k_-Lys-BN.

**Apparent receptor affinity (IC_50_)**

*Material & Methods:* PC-3 cells at confluence were placed in 48-well plates and left to attach overnight. The cells were incubated at 37°C in a special binding buffer (50 mM HEPES, 125 mM NaCl, 7.5 mM KCl, 5.5 mM MgCl_2_·6H_2_O, 1 mM ethylene glycol tetraacetic acid, 5 g/L BSA, 2 mg/L chymostatin, 100 mg/L soybean trypsin inhibitor, 50 mg/L bacitracin, pH 7.4) with increasing concentrations (0-30000 nM) of the unlabelled BN analogues (final volume 0.2 ml/well) in the presence of 4 kBq of ^99m^Tc-BN(7-14), which is known to bind to the GRP receptor with high affinity (K_d_=0.21 nM), as a surrogate for BN(1-14) [3]. After 1 h incubation at 37°C, the cells were twice washed with cold PBS and solubilized by adding 400 μl (2 x) of 1 N NaOH at 37°C. The bound radioactivity was measured in the gamma counter. The experiments were performed in triplicate.

*Results:* Receptor affinity assays showed that the half maximal inhibitory constant (IC_50_) for the DOTA-PEG_5k_-Lys-BN was 15.96±4.42 nM. This value is slightly higher than the IC_50_ value of the non-PEGylated DOTA-Lys-BN (2.03±0.42 nM). Since IC_50_ determination was done on whole PC-3 cells, with which internalisation could happen, the above mentioned values are apparent IC_50_ values, because the binding to the receptor might not be in equilibrium.

**Serum analysis**

*Material & Methods:* Serum analysis was performed with three groups of mice which were included in the ^99m^Tc-DMSA studies (group G-I, Table 1). At day 111 after treatment, blood was drawn from the sublingual vein. The blood was collected in heparinized vials Microvette 500 Lithium-Heparin (Sarstedt, Sevelen, Switzerland) and centrifuged to allow collection of (5 min, 1500 g). Then, ~150 μl serum plasma were withdrawn and stored at -20°C for analysis. Serum urea and creatinine levels were measured with a Cobas Integra 800 analyzer (Roche Diagnostics, Rotkreuz, Switzerland).

*Results:* There was no significant difference between group G, H and I in the serum creatinine and urea levels 111 days after treatment. The control mice, the mice treated with high specific activity and the mice treated with low specific activity all showed serum creatinine levels <18 μmol/L and serum urea values between 6 and 8 mmol/L.

**Preliminary therapy study**

*Purpose:* The effect of the ^177^Lu-DOTA-PEG_5k_-Lys-BN analogue on tumour growth was investigated in a pilot study with only four mice per group and compared to the tumour growth of untreated animals. Furthermore, we wanted to compare the antitumour effect obtained with only one dose at day 0 (1 x 20 MBq per mouse) with the effect obtained after two doses at days 0 and 14 (2 x 20 MBq per mouse).

*Experimental Design and Results:* Preliminary therapy studies were performed in mice bearing PC-3 xenografts. Two weeks post PC-3 inoculation, i.e. the day of the first injection (day 0), the tumours had an average volume measurement of 61 mm^3^. The animals were divided into three groups of four mice each. (1) The control group received PBS i.v. at days 0 and 14. The treated animals received ^177^Lu-DOTA-PEG_5k_-Lys-BN i.v. either (2) in a single dose of 20 MBq (3.0 nmol peptide) at day 0 or (3) in two equal doses of 20 MBq (2 x 20 MBq = 40 MBq, 3.0 nmol peptide each) at days 0 and 14. Upon euthanasia, tumours were collected, embedded in TissueTek (O.C.T. Compound; Sakura Finetek Europe B.V.) and frozen for autoradiography.

The treatment with one or two doses of ^177^Lu-DOTA-PEG_5k_-Lys-BN (20 MBq vs. 40 MBq) significantly decreased the PC-3 tumour growth rate with respect to that of the control group. After applying the ^177^Lu-DOTA-PEG_5k_-Lys-BN analogue only once, the tumour growth was reduced by 60% compared to that of control animals three weeks after the first dose. Applying the ^177^Lu-DOTA-PEG_5k_-Lys-BN analogue twice, the inhibition was even 80%. In comparison to the control, the relative tumour volumes of the treated animals were significantly smaller (*P* < 0.05) from day 7 to day 21 (Supplemental Figure 8).

*Conclusion:* The preliminary therapy studies, in which the number of doses was varied in order to explore the therapeutic potential, showed, as expected, that the administration of two doses (2 x 20 MBq = 40 MBq) was more effective in tumour growth inhibition than administering a single dose (1 x 20 MBq) (Supplemental Figure 8).

**Supplemental Figure 8** Therapeutic effect of ^177^Lu-DOTA-PEG_5k_-Lys-BN in mice with PC-3 tumour xenografts. The graph shows a comparison of tumour growth from animals of group 1 (control), group 2 (1 x 20 MBq) and group 3 (2 x 20 MBq). Data are expressed as volume of tumour relative to the volume in the same animal immediately before the first injection (mean ± SD of 4 animals)

**In vitro autoradiography of tumour sections**

*Purpose:* The administration of two doses (2 x 20 MBq) ^177^Lu-DOTA-PEG_5k_-Lys-BN was more effective in tumour growth inhibition than administering a single dose (1 x 20 MBq) (Supplemental Figure 8). However, the tumours started to grow again within one week after the second dose. It seemed that the second dose was less effective than the first one. In order to rule out the possibility of down-regulation of BN_2_/GRP receptors as a consequence of the application of ^177^Lu-DOTA-Lys-BN analogues, we performed in vitro autoradiography studies on frozen tumour sections collected from treated mice after euthanasia.

*Experimental Design and Results:* For quantification of the BN_2_/GRP receptors, frozen PC-3 tumours from animals of the preliminary therapy study (26 days after the first dose) and from the animals of the main therapy study reported in the main article (21 days after the first dose) were cut into sections of 4 μm (Bright OTF Cryostat, Walldorf, Germany) and mounted on glass slides (Superfrost plus, Menzelgläser, Braunschweig, Germany). The tumour sections were incubated for 1 h in 50 kBq (100 μl) of the ^99m^Tc-labelled BN(7-14), which was prepared according to the usual labelling procedure as previously published [1]. After several washing steps of the frozen tissue sections in buffer (167 mM Tris-HCl, 5 mM MgCl_2_·6H_2_O, 2.5g/L BSA, pH 7.6) and destilled water, the sections were exposed to the phosphor imaging screens (super resolution type SR; PerkinElmer, Schwerzenbach, Switzerland) in x-ray cassettes. After 3 h, the screens were read by the Cyclone phosphor imager and radioactivity bound to tissue samples was quantified using the OptiQuant 3.00 image processing system (PerkinElmer, Groningen, The Netherlands).

The blackening of the sections (DLU/mm^2^) after in vitro incubation with a radioactive BN analogue was quantified and was used as a measure for determining the amount of BN_2_/GRP receptors. For the preliminary therapy study the value obtained for the control mice was set to 100% and did not significantly differ from the value for the treated animals (91%) which indicates that BN_2_/GRP receptors were not down-regulated as a consequence of targeted radionuclide therapy.

The in vitro autoradiography, which was performed with PC-3 tumour sections of control mice (Supplemental Figure 9A) and mice treated with ^177^Lu-DOTA-PEG_5k_-Lys-BN of high or low specific activity (Supplemental Figures 9B and 9C) from the main therapy study reported in the main article, confirmed the results of the preliminary study. The radioactivity bound in tumour sections of the treated animals with high specific activity (84%) was similar to the radioactivity of the treated animals with low specific activity and the control mice (103% and 100%, respectively). This indicates that there was no long-lasting down-regulation of BN_2_/GRP receptors in the tumour after treatment.

*Conclusion:* The fact that a BN tracer has bound to tumour sections of treated or of control mice in similar amounts proved that there was no down-regulation of BN_2_/GRP receptors. This is in agreement with the fast BN_2_/GRP receptor recovery after injection, which was observed in the studies with AMBA and PESIN reported in the literature [4-5]. The second dose would therefore be supposed to have had the same effect as the first dose. However, the fact that the second dose differs from the first dose in its duration of antitumour effect is presumably due to the larger tumour size at the time of the second dose compared to that of the first dose at the beginning of the therapy. Thus, our data confirmed the results of the ^177^Lu-AMBA study, which showed that two doses reduce the tumour growth more effectively and improve overall survival in comparison to treatment with a single dose [4].

**Supplemental Figure 9** In vitro autoradiograms of PC-3 tumours from control mice **a** and mice of the main therapy study (reported in the main article) 7 days after the second dose of ^177^Lu-DOTA-PEG_5k_-Lys-BN with low specific activity **b** and with high specific activity **c**. Left panel: tumour sections incubated with the ^99m^Tc-BN analogue only; right panel: tumour sections co-incubated with ^99m^Tc-BN analogue and non-radioactive BN to block receptors

**References**

[37] Garcia Garayoa E, Rüegg D, Bläuenstein P, Zwimpfer M, Khan IU, Maes V, et al. Chemical and biological characterization of new Re(CO)_3_/[^99m^Tc](CO)_3_ bombesin analogues*.* Nucl Med Biol. 2007;34:17-28.

[38] Pillarsetty N, Cai S, Ageyeva L, Finn RD, and Blasberg RG. Synthesis and evaluation of [^18^F] labeled pyrimidine nucleosides for positron emission tomography imaging of herpes simplex virus 1 thymidine kinase gene expression*.* J Med Chem. 2006;49:5377-81.

[39] La Bella R, Garcia-Garayoa E, Bahler M, Blauenstein P, Schibli R, Conrath P, et al. A ^99m^Tc(I)-postlabeled high affinity bombesin analogue as a potential tumor imaging agent*.* Bioconjug Chem. 2002;13:599-604.

[40] Lantry LE, Cappelletti E, Maddalena ME, Fox JS, Feng W, Chen J, et al. ^177^Lu-AMBA: Synthesis and characterization of a selective ^177^Lu-labeled GRP-R agonist for systemic radiotherapy of prostate cancer*.* J Nucl Med. 2006;47:1144-52.

[41] Wild D, Frischknecht M, Zhang H, Morgenstern A, Bruchertseifer F, Boisclair J, et al. Alpha- versus beta-particle radiopeptide therapy in a human prostate cancer model (^213^Bi-DOTA-PESIN and ^213^Bi-AMBA versus ^177^Lu-DOTA-PESIN)*.* Cancer Res. 2011;71:1009-18.
